# Association of severe depression with bone mineral content: a mediation analysis in PERSIAN cohort study

**DOI:** 10.1186/s12889-026-26261-3

**Published:** 2026-01-29

**Authors:** Mahdieh Sahebi, Mahla Daliri, Fateme Nikbakht, Samaneh Eslami, Mohammad H. Ebrahimzadeh, Ali Moradi, Ali Taghipour, Masoumeh Sadeghi

**Affiliations:** 1 Department of Epidemiology, Faculty of Health, Mashhad University of Medical Sciences, Mashhad, Iran; 2 Orthopedics Research Center, Ghaem Hospital, Mashhad University of Medical Sciences, Mashhad, Iran; 3 Torbat Jam University of Medical Sciences, Torbat Jam, Iran; 4 Social Determinants of Health Research Center, Mashhad University of Medical Sciences, Mashhad, Iran

**Keywords:** Bone mineral, Depression, Psychology, Mediation analysis, PERSIAN cohort

## Abstract

**Background:**

Depression may lead to lower Bone Mineral Content (BMC), as an indicator of bone health. In this study, we aim to estimate the direct, indirect, and total associations of depression with BMC through various mediators to explain the mechanism behind the reduction in BMC.

**Methods:**

This is a cross-sectional study within the PERSIAN perspective cohort study at Mashhad University of Medical Sciences (MUMS) in northeastern Iran, involving 3179 personnel in 2019–2020. Depression was assessed using the standard DASS-21 questionnaire. Smoking, taking sleeping pills, falling asleep duration, and metabolic equivalents (METs) served as mediators, based on the literature review. We employed mediation analysis packages, with a counterfactual approach, to estimate the various associations between depression, the mediators, and BMC using Stata.

**Results:**

The mean age of participants with moderate and severe depression levels was 43.26 and 42.59 years, respectively. Most of the participants with depression were women. In multivariable analysis, the reference interaction (coef = 0.012), mediated interaction (coef = 0.004), and controlled direct effect (coef = -0.024) of severe depression through the falling asleep duration, as well as the pure indirect effect of severe depression through METs (coef = 0.001), remained significant (*p* < 0.05).

**Conclusion:**

Severe depression level demonstrates both direct and indirect associations with BMC, along with interactions with smoking and the falling asleep duration as mediators. All the mediators exhibit a pure direct association with BMC. Nevertheless, it appears that our confounding variables exert a strong influence on these relationships, as most of the associations were not significant after adjustment.

**Supplementary Information:**

The online version contains supplementary material available at 10.1186/s12889-026-26261-3.

## Background

The importance of preventing osteoporotic fragility fractures is growing as the global population ages. In the United States, osteoporotic fractures are exceedingly common; an estimated 1.5 million people experience fragility fractures annually [1]. Bone Mineral Content (BMC) and Bone Mineral Density (BMD) are related to mental disorders, such as major depressive disorder (MDD), mediated by poor lifestyle habits, sympathetic nerve stimulation, and certain immunological and endocrine processes associated with depression [2, 3]. Higher depressive symptoms were associated with lower total body BMC and BMD [4]. Various studies showed that depression was associated with smoking, sleep duration and sleep difficulties, use of sleeping pills, and alcohol consumption [4–8]. Also, several studies showed the association between bone health and alcohol consumption, smoking, and metabolic equivalents (METs) [4, 9, 10]. Bone health and fracture risk depend on established predictors such as age, sex, BMI, prior fractures, glucocorticoid use, rheumatoid arthritis, secondary osteoporosis, smoking, and alcohol intake. The FRAX tool integrates these clinical risk factors to estimate fracture probability, with or without bone mineral density measurements. In studies linking depression and bone health, these factors may confound or mediate associations [11]. Therefore, accounting for these predictors is essential to avoid bias in interpreting bone health outcomes. These factors can be mediator variables in the association between depression and BMC, for example, regular users of both cigarettes and alcohol demonstrated a stronger negative association between depressive symptoms and BMC compared with non-users/experimental users and regular alcohol users [4]. However, despite growing literature on this topic, there is no comprehensive study on the impact of mediators and cofounders to help better understand the mechanisms and risk factors for the lower BMC among people with depression.

Most previous studies have employed conventional regression models to examine the association between depression and bone mineral content (BMC). However, such approaches do not fully disentangle the complex pathways through which depression may influence BMC. Therefore, advanced statistical methods like mediation analysis with a counterfactual framework are recommended, as they allow for estimation of the direct effect of depression on BMC, the indirect effects mediated through other biopsychosocial factors, and the interaction effects between depression and these mediators. In this study, we hypothesized that severe depression is associated with a reduction in BMC directly, and indirectly via increased smoking and sleeping pill use, prolonged falling asleep duration, and decreased physical activity (measured by MET).

## Methods

### Study setting and ethical considerations

This is a cross-sectional study within the PERSIAN Organizational Cohort Study in MUMS (POCM), conducted at Imam Reza Hospital, involving 3179 participants with completed Dass-21 questionnaire of total of 4737 personnel affiliated with Mashhad University of Medical Sciences (MUMS) in 2019–2020. One thousand five hundred fifty-eight participants were excluded from the study because of insufficient data about the Dass-21 questionnaire (Supplementary 1). Validity and reliability have previously been tested in Iran. This questionnaire contains 21 items including 8 related to depression (D), 7 items related to anxiety (A), and 6 items related to stress (S). In this test, individuals are asked to mark their states over the past week based on the statements provided in the questionnaire, considering four specified categories. The response form is arranged in four verbal categories: (not at all), (somewhat), (much), and (very much), and is completed by all 174 sample individuals. If any statements are unclear to the respondents, explanations are provided by the researcher.

**Fig. 1:**
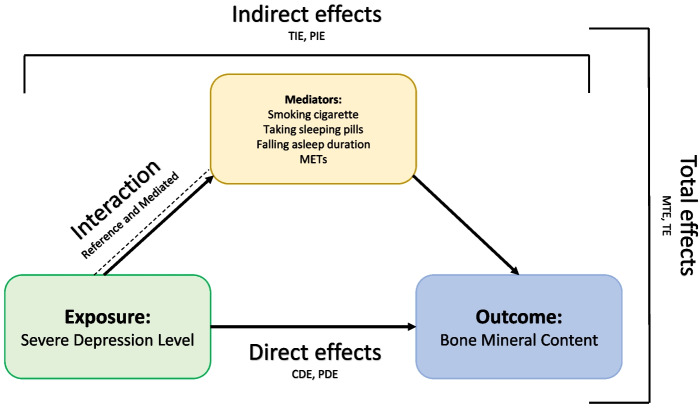
Direct and indirect effects of severe depression level on bone mineral content through multiple mediators. The figure illustrates the pathways of total effects (TE), decomposed into direct effects (controlled direct effect [CDE] and pure direct effect [PDE]) and indirect effects mediated by smoking cigarettes, taking sleeping pills, falling asleep duration, and metabolic equivalent tasks (METs). Interaction between exposure and mediators is also depicted

The Cronbach’s alpha coefficient was acceptable for anxiety (0.79), stress (0.91), and depression (0.93). An acceptable test–retest reliability (0.740–0.881, *P* < 0.01) was also reported for DASS-21 and its three dimensions [12]. The Institutional Human Research Review Board of the MUMS has approved the study protocol and research ethics (IR.MUMS.MEDICAL.REC.1401.496). The present research adhered to the principles articulated in the Helsinki Declaration. Before participation, all individuals provided informed consent and retained the right to withdraw from the study at any point. Detailed descriptions regarding the participants' enrollment process in the PERSIAN cohort and study design have been reported elsewhere [13] and depicted in Fig. 1.

Trained research nurses proficient in data collection protocols conducted interviews and assessments to obtain both nonclinical and clinical data, encompassing demographic characteristics, sleep patterns, circadian rhythm, and habits (e.g., smoking). A description of the data collection protocol is available in our previous publications [14].

### Mediator variables

Smoking cigarettes (current smoking), taking sleeping pills, the falling asleep duration and Metabolic Equivalent of Task (MET) were mediators in our study. These mediators are chosen based on a literature review. Depression is linked with smoking habits, which adversely affect bone health. Sleep quality and disturbances, taking sleep medications, are prevalent in individuals with depression and can impact bone metabolism. Physical activity, which shows a reduction in participants with depression, contributes to bone loss [15–30] and the availability of data. MET, as a continuous variable, was employed to assess the intensity of physical activity. One MET is equivalent to the energy expenditure of quiet sitting, equivalent to approximately 3.5 mL of oxygen uptake per kilogram of body weight per minute. Participants reported the duration of various activities, including sleep duration, daytime rest, awake time in bed, television viewing, study time, desk work, computer usage, driving, eating, cooking, house cleaning, sales activities, walking, aerobic exercise, driving heavy vehicles, light technical work, masonry, carrying light objects, light agriculture, heavy agriculture, heavy engineering, and intense physical exercise. Each activity was assigned a standardized MET value according to the Compendium of Physical Activities, reflecting its relative intensity. The total MET score was calculated by multiplying the MET value of each activity by the time spent performing that activity, then summing these values across all activities. This provided a continuous measure of overall physical activity level and intensity [31].

### Confounding variables

A thorough literature review [32–34] and clinical relevance assessment were conducted. BMI, gender, age, marital status, education degree, ethnicity, calcium, protein, diabetes, hypertension, Rheumatic disease, psychiatric disorders, and taking drugs were checked using univariate linear regression for the confounding role. BMI, gender, age, marital status, education degree, calcium, protein, hypertension, and psychiatric disorders with a significant level of < 0.25 were included in multivariable analysis.

Measurement Tools:

- InBody: BMC was measured using the InBody 770 device, which employs Bioimpedance analysis based on DSM-BIA1 technology. This device administers a weak alternating electric current (50–1000 kHz) through the body, with different tissues reflecting varying portions of the waves in accordance with their tissue type. The device quantifies the reflected waves and subsequently calculates the density of different body masses.
- DASS-21 Questionnaire: Depression was assessed using the DASS-21 questionnaire, which comprises seven questions addressing dysphoria, hopelessness, devaluation of life, self-deprecation, lack of interest/involvement, anhedonia, and inertia [35]. The Cronbach's alpha coefficient for depression was acceptable at 0.93. Additionally, an acceptable test–retest reliability ranging from 0.740 to 0.881 (*P* < 0.01) was reported for the DASS-21 and its three dimensions [12]. A binary exposure (moderate depression and severe depression) was determined based on the severity of depression using a predefined cut-off point. A score less than or equal to 20 indicates moderate depression, and the score equal to or more than 21 is considered severe depression [36].

### Statistical analysis

Descriptive statistics were reported by frequency (%) for categorical variables and mean and standard deviation for continuous variables. Univariate and multivariable mediation analysis was performed using the PARAMED and MED4WAY packages in Stata, employing a counterfactual approach. PARAMED estimated the controlled direct effect (CDE), pure direct effect (PDE), total indirect effect (TIE), and marginal total effect (MTE) [37]. We evaluated mediation and interaction using the counterfactual four-way decomposition proposed by VanderWeele, which partitions the total effect into four components: the controlled direct effect, the reference (pure) interaction, the mediated interaction, and the pure indirect (mediation) effect. This decomposition is particularly useful when the exposure may both change the value of a mediator and modify how that mediator affects the outcome; it therefore separates mediation from interaction on the additive scale and quantifies the contribution of each pathway. According to VanderWeele (2014), the controlled direct effect (CDE) and the pure direct effect (PDE) both represent pathways by which an exposure affects an outcome independently of mediation, but they differ in how the mediator is handled. The CDE estimates the effect of the exposure on the outcome when the mediator is artificially fixed at a specific value for all individuals, thereby allowing exposure–mediator interaction to influence the effect size. In contrast, the PDE estimates the effect of the exposure when the mediator is set to the level it would naturally take in the absence of exposure, thus isolating the direct effect that is free from both mediation and exposure–mediator interaction. Consequently, the CDE reflects a hypothetical intervention fixing the mediator, whereas the PDE represents the natural direct effect under no exposure. These packages entailed two regression models, one for the mediator and the other for the outcome, from which mediation effects were derived. Depression, smoking, and taking sleeping pills were considered binary variables, while bone mineral content, sleep duration, and MET were considered continuous variables. Mediation effect coefficients were reported with 95% confidence intervals, and *p*-values < 0.05 were considered statistically significant. The assumptions of mediation analysis, including the absence of unmeasured exposure-outcome, mediator-outcome, exposure-mediator, and mediator-outcome confounders influenced by the exposure, were assessed, and any identified confounders were incorporated into multivariable (or adjusted) models. Stata version 14 (Stata Corp., College Station, TX, USA) was employed to conduct all statistical analyses.

## Results

Participants with moderate and severe depression levels had a mean age of 43.26 and 42.59, respectively. Married women made up the majority of participants in both types of depression severities. The most common degrees held by participants were bachelor's and master's. Out of the total participants, 2812 exhibited moderate depression, while 367 had severe depression. Notably, a majority of individuals in both depression categories were female and married (Table 1).

**Table 1:** The study population descriptive data (*N* = 3179)

*Variable*		Depression		*P*-value^1^
		Moderate depression (*N* = 2812)	Severe depression (*N* = 367)	
*Age (mean* ± *SD)*		8.42 ± 43.26	7.91 ± 42.59	0.1487
*Gender N (%)*	*Men*	47.26))1329	(40.05)147	0.009*
	*Women*	52.74))1483	(59.95)220	
*BMI (mean* ± *SD)*		4.72 ± 26.91	16.46 ± 27.98	0.5135
*Marital Status N (%)*	*Single*	234 (8.32)	32 (8.72)	0.010*
	*Married*	2450 (87.13)	305 (83.11)	
	*Widow or divorce*	128 (5.55)	30 (8.17)	
*Educational level N (%)*	*Undergraduate*^*2*^	356 (12.66)	78(21.25)	0.000*
	*Postgraduate*^*3*^	1749 (62.20)	224(61.04)	
	*PhD*	707 (25.14)	65 (17.71)	
*Smoking status*	*Yes*	2598(93.05)	334(91.26)	0.210
	*No*	194(6.95)	32(8.74%)	
*Sleep duration (Nighttime sleep) (mean ± SD)*		6.82 ± 0.02	6.80 ± 0.08	0.897
*BMC (mean ± SD)*		2.78 ± 0.01	2.68 ± 0.01	0.005*

^1^*p*-value < 0.05

^2^including illiterate, elementary, middle school, and diploma

^3^including bachelor and master

* denotes a statistical significance level *p*<0.05

In the univariate mediation analysis, severe depression exhibited a direct effect through several pathways: smoking (coefficient = −0.107, *p* = 0.001), taking sleeping pills (coefficient = −0.08, *p* = 0.009), the duration of falling asleep (coefficient = −0.06, *p* = 0.044), MET (coefficient = −0.09, *p* = 0.003), and a reference interaction with smoking (coefficient = −0.218, *p* = 0.043). Controlled direct effects of severe depression were observed when the mediators were taking sleeping pills (coefficient = −0.11, *p* = 0.006) or the duration to fall asleep (coefficient = −0.11, *p* = 0.012). Additionally, sleeping pill use (coefficient = −0.017, *p* = 0.006), falling asleep duration (coefficient = −0.017, *p* = 0.002), and MET (coefficient = 0.010, *p* = 0.033) had a pure indirect effect on Bone Mineral Content (BMC). The overall impact of severe depression through smoking, falling asleep duration, and MET on BMC was statistically significant (coefficient = −0.09, *p* < 0.05) (Table 2), 3). The total effect of severe depression through all mediators was significant in the MED4WAY package (Table 3). Notably, all these coefficients contributed to a decrease in bone mineral content, except for MET.

**Table 2:** Mediation analysis of depression as the exposure and bone mineral content as the outcome with PARAMED package

Mediator	effect	Crude analysis^1^			Adjusted analysis^2^		
		R^3^	CI^4^	*P* value	R	CI	*P* value
Smoking cigarette	CDE^5^	0.11	(−0.10,0.32)	0.316	0.000	(−0.04,0.05)	0.983
	PDE^6^	−0.10	(−0.17, −0.04)	0.001*	−0.009	(−0.02,0.00)	0.212
	TIE^7^	0.01	(−0.00,0.03)	0.255	0.000	(−0.00,0.00)	0.346
	MTE^8^	−0.09	(−0.16, −0.02)	0.006*	−0.009	(−0.02,0.00)	0.243
taking sleeping pills	CDE	−0.09	(−0.16, −0.02)	0.006*	−0.010	(−0.02,0.00)	0.200
	PDE	−0.08	(−0.15, −0.02)	0.009*	−0.009	(−0.02,0.00)	0.214
	TIE	−0.00	(−0.01,0.01)	0.960	0.000	(−0.00,0.00)	0.492
	MTE	−0.08	(−0.18,0.00)	0.058	−0.008	(−0.06,0.04)	0.742
Falling asleep duration (min)	CDE	−0.11	(−0.20, −0.02)	0.012*	−0.024	(−0.04, −0.00)	0.023*
	PDE	−0.06	(−0.13, −0.00)	0.044*	−0.012	(−0.00,0.00)	0.125
	TIE	−0.02	(−0.04, −0.00)	0.038*	0.003	(−0.00,0.00)	0.093
	MTE	−0.09	(−0.15, −0.025)	0.038*	−0.008	(−0.02,0.00)	0.246
MET	CDE	−0.34	(−0.70,0.02)	0.067	0.003	(−0.07,0.08)	0.925
	PDE	−0.09	(−0.16, −0.03)	0.003*	−0.010	(−0.02,0.00)	0.161
	TIE	0.005	(−0.00,0.01)	0.084	0.002	(−0.00,0.00)	0.055
	MTE	−0.09	(−0.15, −0.02)	0.005*	−0.008	(−0.02,0.00)	0.263

^1^Crude Analysis: Mediation analysis without confounders

^2^Adjusted Analysis: Mediation Analysis controlling for BMI, gender, age, marital status, education degree, calcium, protein, hypertension, and psychiatric disorders as confounders

^3^R = coefficient

^4^CI = confidence interval

^5^CDE = controlled direct effect

^6^PDE = pure direct effect

^7^TIE = total indirect effect

^8^MTE = marginal total effect

* denotes a statistical significance level *p*<0.05

**Table 3:** Mediation analysis of depression as exposure and bone mineral content as outcome with MED4WAY package

Mediator	Effect	Crude analysis^1^			Adjusted analysis^2^		
		R^3^	CI^4^	*P* value	R	CI	*P* value
Smoking cigarette	TE^5^	−0.09	(−0.16, −0.02)	0.007*	−0.009	(−0.02,0.00)	0.228
	CDE^6^	0.11	(−0.10,0.32)	0.315	0.000	(−0.04,0.05)	0.983
	INTREF^7^	−0.21	(−0.42, −0.00)	0.043*	−0.01	(−0.06,0.04)	0.695
	INTMED^8^	0.004	(−0.00,0.01)	0.299	0.000	(−0.00,0.00)	0.704
	PIE^9^	0.009	(−0.00,0.02)	0.230	0.000	(−0.00,0.00)	0.230
taking sleeping pill	TE	−0.08	(−0.15, −0.02)	0.007*	−0.008	(−0.02,0.00)	0.242
	CDE	-.095	(−0.16, −0.2)	0.006*	−0.01	(−0.02,0.00)	0.200
	INTREF	0.006	(−0.00,0.01)	0.056	0.000	(−0.00,0.00)	0.363
	INTMED	0.01	(−0.00,0.03)	0.074	0.001	(−0.00,0.00)	0.373
	PIE	−0.01	(−0.03,−0.00)	0.006*	−0.000	(−0.00,0.00)	0.556
Falling asleep duration (min)	TE	−0.08	(−0.15, −0.02)	0.007*	−0.008	(−0.02,0.00)	0.245
	CDE	−0.11	(−0.16, −0.02)	0.006*	−0.02	(−0.04, −0.00)	0.023*
	INTREF	0.006	(−0.00,0.01)	0.058	0.01	(0.00,0.02)	0.029*
	INTMED	0.01	(−0.00,0.03)	0.059	0.004	(0.00,0.00)	0.039*
	PIE	−0.01	(−0.02, −0.00)	0.002*	−0.001	(−0.00,0.00)	0.187
MET	TE	−0.09	(−0.15, −0.02)	0.005*	−0.008	(−0.02,0.00)	0.262
	CDE	−0.34	(−0.70,0.02)	0.067	0.003	(−0.09,0.06)	0.925
	INTREF	0.24	(−0.12,0.60)	0.193	−0.01	(−0.00,0.00)	0.729
	INTMED	−0.004	(−0.01,0.00)	0.260	0.000	(−0.00,0.00)	0.731
	PIE	0.01	(0.00,0.01)	0.033*	0.001	(0.00,0.00)	0.021*

^1^Crude Analysis: Mediation analysis without confounders

^2^Adjusted Analysis: Mediation Analysis controlling for BMI, gender, age, marital status, education degree, calcium, protein, hypertension, and psychiatric disorders as confounders

^3^R = coefficient

^4^CI = confidence interval

^5^TE = total effect

^6^CDE = controlled direct effect

^7^INTREF = reference interaction

^8^INTMED = mediated interaction

^9^PIE = pure indirect effect

* denotes a statistical significance level *p*<0.05

In the multivariable mediation analysis, the controlled direct effect coefficient of severe depression through the falling asleep duration was −0.024 (CI: −0.04 to −0.00). Both types of reference interaction (coefficient = 0.012, CI: 0.00 to 0.02) and mediated interaction (coefficient = 0.004, CI: 0.00 to 0.00) between severe depression and the falling asleep duration were statistically significant. The pure indirect effect coefficient of MET was 0.001 (CI: 0.00 to 0.00) (see Fig. 1).

## Discussion

This study employed mediation analysis using a counterfactual approach to examine the association between severe depression and bone mineral content (BMC), mediated by cigarette smoking, sleeping pill use, falling asleep duration, and metabolic equivalent of task (MET). The controlled direct effect (CDE) of severe depression on BMC via falling asleep duration indicates that fixing this mediator at a specific value reduced BMC by 0.024. In contrast, the reference interaction between severe depression and falling asleep duration increased BMC by 0.012 (95% CI: 0.00 to 0.02), even after preventing severe depression as the exposure. The mediated interaction further raised BMC by 0.004 (95% CI: 0.00 to 0.00), requiring both mediation and interaction effects for interpretation—unlike pure indirect effects, which isolate the mediator's role when exposure solely induces it. The pure indirect effect of MET modestly increased BMC by 0.001 (95% CI: 0.00 to 0.00).

Our finding of an adverse association aligns with recent epidemiological syntheses and cohort studies that report lower bone mass and an elevated fracture risk among individuals with depressive disorders. Although the magnitude and site-specific effects vary across studies, the overall pattern of a negative relationship between depression and bone health is well documented. Behavioral mediators, such as reduced weight-bearing activity, smoking, and poor sleep, are plausible contributors and have been implicated in previous observational and mechanistic studies [38]. Biologically, depression can impact bone metabolism through the dysregulation of the hypothalamic–pituitary–adrenal axis, chronic inflammation, and sympathetic activation [39]. Depression is behaviorally characterized by factors such as physical inactivity, smoking, and sleep dysfunction, all of which are strongly linked to lower bone mineral density (BMD) [40, 41].

Many studies have reported the overall impact of depression on bone health. For example, a study investigating the association between depression and anxiety symptoms and bone health found that severe depressive symptoms are associated with lower bone mineral content (BMC) [4]. Another study investigating the influence of alcohol consumption, place of residence, and smoking on BMC revealed that smoking cigarettes had a weak but significant impact on BMC [9]. Recent longitudinal analyses reported that Lifestyle factors like cigarette smoking showed a modest but significant effect on BMD [42, 43]. However, the results of the multivariable analysis in our study did not show a significant relationship between smoking cigarettes and BMC. Nevertheless, in the univariate analysis, the pure direct effect of smoking cigarettes was found to reduce BMC.

Additionally, Abolhassani et al. indicated a higher prevalence of sleeping pill use in individuals with depression compared to those without depression [44]. The association between severe depression and BMC through the use of sleeping pills was not statistically significant in our multivariable analyses. However, in the univariate analysis, the pure indirect effect of taking sleeping pills was significant and contributed to a decrease in BMC. Differences in study populations, analytical approaches, and measurement methods between our study and Abolhassani's study may explain the varying results. Nevertheless, these findings suggest that severe depression may lead to a decrease in BMD through the use of sleeping pills. A recent updated meta-analysis also found no significant difference in BMD between men with major depressive disorder (MDD) and control groups after stratifying by mean age, gender, recruitment, diagnostic criteria, and measurement techniques. Their findings indicate that the association between BMD and depression is site-specific, with a decrease in BMD more likely to occur in the spine, hip, and femoral neck among individuals with MDD, but not in the forearm or femoral trochanter. Additionally, this relationship is influenced by gender; in gender-stratified analyses, MDD, which can lower BMD, was significantly more prevalent in women than in men. [45]. Generally, these findings indicate that both behavioral and pharmacological responses may partially mediate the association between depression and bone health, and that these effects vary by skeletal site and sex.

Moreover, a study by Wei Lin et al. demonstrated that pregnant individuals with depression encountered difficulties in falling asleep (Odds Ratio = 6.04, 95% CI: 4.19 to 8.72) [46]. Sandra Pedraza's work reported that high depressive symptoms are associated with trouble falling asleep [47]. Nobuo Sasaki's study also revealed that poor sleep quality is linked to bone stiffness [48]. In our current study, the Controlled Direct Effect (CDE) and Pure Indirect Effect (PIE) of severe depression on BMC through the duration of falling asleep showed an association between depression, falling asleep duration, and BMC, respectively, aligning with recent studies that poor sleep quality predicts low BMD and increased risk of fracture [49]. Additionally, severe depression and falling asleep duration exhibited an interaction, leading to an increase in BMC. At first, this result may appear paradoxical, because most studies reported poor sleep quality, prolonged sleep latency are risk factors for lower BMD [22, 24, 48]. A possible explanation is that individuals with severe depression may exhibit compensatory sleep patterns, such as longer or adequate nighttime sleep or earlier sleep onset. Under certain circumstances, these patterns can improve bone metabolism through hormonal rhythms. This theory is supported by studies indicating that adequate sleep contributes to better bone health by enhancing the secretion of growth hormones and regulating the hypothalamic–pituitary–adrenal (HPA) axis [50, 51]. Prior studies showed that participants with severe depression may use sleep aids, which lead to longer sleep duration and better rest, which indirectly improves bone health [15, 44]. Bone mineral content changes are typically modest, and even 1–2% differences in bone mass have been associated with substantial changes in fracture risk at the population level. Therefore, the observed interaction effects, while numerically small, are clinically relevant in the context of skeletal health [52].

Furthermore, research aiming to investigate the determinants of bone health reported that the MET score increased Bone Mineral Density (BMD) by 0.163 (*p* = 0.026) [10]. Physical activity improves balance and muscle mass, which reduces fractures and skeletal disorders even in individuals with lower BMD [43]. Additionally, an inverse association between MET and depression was observed in Kim et al.'s study, suggesting that MET may reduce the onset of depressive symptoms, supporting the hypothesis that activity improves mental health besides bone health [53]. In our study, the pure indirect effect of MET on BMC resulted in a small increase in BMC, which shows the protective effect against the negative impact of severe depression. This result is consistent with a longitudinal study, which indicated that even modest increases in physical activity can decrease bone loss in individuals with depression [54].

Our study population, personnel of Mashhad University of Medical Sciences, experience occupational stress, long work hours, and frequent rotating shifts, which predispose them to depression, low sleep quality, and unfavorable lifestyle patterns. A recent meta-analysis reported high depression prevalence among Iranian nurses [55]. Also, night workers show a significant reduction in BMD [56], especially among women. These findings raise concerns about bone and skeletal health in these workers. Additionally, women, especially in midlife, experience stronger associations between depression and bone loss [57]. These factors underscore the need for preventive strategies by gender and occupational profile.

This study benefits from a large sample size and counterfactual mediation analysis, which provide precise effect estimates.

However, several limitations warrant caution. The cross-sectional design precludes causal inference. Severe depression assessment relied solely on the validated DASS-21 self-report questionnaire without structured clinical interviews, potentially introducing information bias and reducing clinical precision. Exclusion of 32.9% of the original sample due to incomplete DASS-21 data may introduce selection bias if missingness was not completely at random, despite similar characteristics between included and excluded participants.

Additional limitations include participants' health status homogeneity, which restricted BMC variation; subjective mediator data collection; single-mediator models to avoid overadjustment and causal ordering violations amid interrelations and unclear temporality (multi-mediator approaches merit future longitudinal exploration); and residual confounding despite extensive covariate adjustments, exacerbated by self-reported mediators. FRAX omission aligned with our BMC focus on fracture risk, but limited accounting for fall-related and psychosocial confounders. Findings from the Mashhad University of Medical Sciences personnel constrain generalizability to broader populations.

## Conclusion

The controlled direct effect, pure indirect effect, reference interaction, and mediated interaction indicate different pathways between severe depression as the exposure and BMC as the outcome through different mediators. Notably, the interaction between severe depression and falling asleep duration was found to increase BMC, underscoring the complexity of behavioral, biological, and pharmacological mechanisms. These findings suggest the presence of distinct pathways and mechanisms contributing to the reduction in BMC. Mediation analysis with counterfactual approach has proven to be a valuable methodological framework beyond traditional regression models. By identifying potential mediating and modifying pathways, our study supports the need for prevention strategies like workplace health programs that consider both mental and lifestyle-related determinants of bone health. Encouraging regular physical activity, improving sleep quality, and offering support for managing depressive symptoms may help preserve bone health, particularly among female staff. Future longitudinal and intervention studies that use objective tools to measure sleep and physical activity with gold-standard assessments of bone health are needed to confirm these pathways whether improving these factors can help prevent bone loss linked to depression.

## Supplementary Information

Supplementary Material 1.

## Data Availability

The datasets used and analysed during the current study are available from the corresponding author on reasonable request.

## Abbreviations

BMC: Bone mineral content
BMD: Bone mineral density
MDD: Major depressive disorder
MET: Metabolic equivalents
CDE: Controlled direct effect
PDE: Pure direct effect
TIE: Total indirect effect
MTE: Marginal total effect
TE: Total effect
INT REF: Reference interaction
INT MED: Mediated interaction
PIE: Pure indirect effect
